# A social network analysis to explore collaborative practice in home care: research protocol

**DOI:** 10.1186/s12913-022-08548-4

**Published:** 2022-09-19

**Authors:** Chloé Schorderet, Caroline H. G. Bastiaenen, Henk Verloo, Robert A. de Bie, Lara Allet

**Affiliations:** 1 School of Health Sciences, HES-SO Valais-Wallis, University of Applied Sciences and Arts Western Switzerland, Valais, Sion, Switzerland; 2 Department of Epidemiology, Care and Public Health Research Institute (CAPHRI), Maastricht University, Maastricht, Netherlands; 3 The Sense, Innovation & Research Center, Sion, Switzerland; 4 Service of Old Age Psychiatry, Lausanne University Hospital, Lausanne, Switzerland; 5 Department of Medicine, University Hospitals of Geneva and University of Geneva, Geneva, Switzerland

**Keywords:** Home care, Social network analysis, Collaborative practice, Home caregivers

## Abstract

**Background:**

The conceptualization of the home as a care environment and maintaining a high standard of care requires different professionals to collaborate. This study will explore collaborative practice in home care, needs and expectations of the stakeholders involved, and identify their roles and tasks. Secondly, it will investigate possible strategies to improve home care management and, more particularly, optimize collaborative practice in home care.

**Methods:**

The study will be conducted during three distinct consecutive phases, within a multiphase mixed-methods design. *Phase 1* will use a quantitative approach in which a social network analysis will be conducted to have an overview of collaborative practice in home care in French-speaking Switzerland. *Phases 2* and *3* will be qualitative and focus on three different situations involving different locations (rural and urban) and different home care functioning (home care provided by agencies and home care providing by independent caregivers). In each situation, semi-structured interviews will be conducted with home care recipients and their home caregivers. In *phase 2*, results of *phase 1*’s network analysis will be discussed, such as roles, needs, and expectations of all stakeholders involved in home care. In *phase 3*, *phase 2*’s findings will be discussed and strategies to improve home care and to optimize collaborative practice will be explored.

**Discussion:**

Over the past years, home care has grown considerably. Therefore, more and more different caregivers are involved in the recipients' homes. Since optimal coordination between these different caregivers is a prerequisite for quality and safe care, it is essential to investigate the existing collaborative practice and how it is functioning. This study will provide knowledge on roles, needs and expectations of different caregivers involved in home care. It will also allow for strategies to optimize collaborative practice and thus ensure comprehensive care for recipients. Finally, it will serve as a basis for future studies that can be conducted to address identified needs.

**Supplementary Information:**

The online version contains supplementary material available at 10.1186/s12913-022-08548-4.

## Background

Europe’s elderly population has been increasing for several decades and projections show that this phenomenon continues in the future [1]. In Switzerland, in 2020, life expectancy at birth was 85.1 years for women and 81.0 years for men [2], of which 71.7 and 70.7 years should be lived in relatively healthy conditions, respectively [3]. Although people now in general live longer and in better health than a few decades ago, there has been a significant increase in the prevalence of chronic and degenerative diseases over the age of 65 [4, 5]. In Switzerland, about half of all people aged 55 years and over and two thirds of those aged 75 years and over live with at least one chronic disease [6]. Of the very old suffering from chronic diseases, around half have two or more chronic diseases, defined as multimorbidity [6]. The latter is particularly responsible for functional decline and disability [7–9]. In this context, the number of complex cases to be managed has increased [10]. Due to the continuing demographic transition, the number of dependent older adults [OA] requiring long-term care is expected to rise further in the coming years [11].

Efficiently and effectively addressing the rise of chronic diseases and multimorbidity is one of the major challenges facing Switzerland’s health system [12]. One solution for coping with this development and addressing patients’ long-term care needs requires a shift from hospitalized / institutionalized rehabilitation to home care [HC] [13].

The literature documents different definitions of HC depending on the study, country, or researchers. This study adopts the definition of WHO Europe, which states that “home care can be conceived of as any care provided behind someone’s front door or, more generally, referring to services enabling people to stay living in their home environment” [14].

With an ageing population and more patients diagnosed with multiple chronic conditions, HC’s importance is evolving. HC has expanded significantly in Europe in recent years [15]. To illustrate the Swiss situation, the number of people aged 65 and over receiving HC services in Switzerland increased from almost 122,000 in 2007 to over 245,000 in 2020 [16]. In 2019, people aged 80 and over received an average of 76 h of professional HC per year, or 1 h 28 min of HC per week [17]. This development of HC is not unexpected in view of its many advantages. First, patient care occurs in a familiar setting. Most OAs needing moderate care over the long term want to be treated in the reassuring environment of their home [18]. In addition, HC is often a good alternative to hospitalization, as the literature demonstrates that this is not always well tolerated, even leading to hospital-related disability [19–22]. This has been shown to be the case for some COPD patients [23], OA stroke patients [24], and patients with acute decompensatory heart failure [25].

Finally, HC makes sense from an economic perspective for the health system. Gonzalez-Jaramillo et al. showed that in addition to reducing the number and duration of hospital visits, and thus, clearly, hospitalization costs, HC reduced overall health care costs in both oncological and non-oncological patients [26]. Di Pollina et al. reported that integrated HC reduces unnecessary hospitalizations and emergency visits among frail elderly patients [27]. Wong et al. reported that a home-based palliative care program was a more cost-effective option than usual palliative care for people with heart failure [28], and Cartoni et al. found similar results, reporting that palliative HC costs were lower than hospital care costs for patients with hematological malignancies [29].

The significant development of HC in recent years has been accompanied by an evolution in its meaning [30]. Professional or policy perspectives generally dominate thinking about HC, meaning that its operational aspects are emphasized [31]. In contrast, the definition of HC preferred by patients and formal and informal caregivers focuses on broader aspects of life, like emotional needs, quality of life, and autonomy [31]. This more holistic approach has gained traction in recent years [31–33], particularly patient-centered care approaches [34, 35] based on each patient’s individual needs [36], including a consideration of the emotional, psychosocial, environmental, and cultural aspects of HC [31]. The development of HC has also been accompanied by an increase in the number of professionals involved. Today more and more stakeholders, with various roles, are active in HC, resulting in the parallel development of the concepts of collaborative practice, patient preferences, and care management. In addition, information, and communication technologies [37–39] have further influenced HC and now offer solutions facilitating care delivery, collaborative practice, and training of future professionals.

Coordination and collaboration between different stakeholders intervening in recipients’ homes is a fundamental element of HC’s evolution [40–45]. A recent scoping review highlighted that interaction and communication between health care professionals were key factors in optimizing HC [46]. Transparent, flexible communication and collaboration between professionals, but also between professionals and care recipients and their families, seem to be necessary ingredients for successful HC [47]. Research has shown that collaborative practice (when different health professionals work with a patient, family, caregivers and the broader health care community) in particular can improve health outcomes for people with chronic diseases, improve overall patient care, and increase patient and caregiver satisfaction [48].

However, achieving good coordination, collaboration, and communication between home caregivers [HCGs] with different care cultures (including different professions) remains a challenge—a challenge that is even greater when it involves treating complex cases with multiple pathologies requiring interventions by several HCGs [10, 49, 50].

The difficulty of coordination and collaborative practice might even be more important between HCGs working independently and not employed by HC agencies. One literature review identified the lack of coordination existing in various European countries when different types of HCGs cared for the same patient in their home [51]. In Switzerland, a study of palliative HC even reported that coordination between services and collaborative practice between specialists and general practitioners were poorly developed [52]. One of the elements that may create tensions and hinder communication and collaborative practice between different stakeholders is their lack of mutual knowledge or misunderstandings about each other’s various professional roles, especially because these have constantly progressed and evolved during the last decade. Yet a clear understanding of each professional’s role in HC management and an in-depth knowledge of their skills from all sides, is fundamental to reinforcing existing collaborative practice and establishing new collaborations that will enhance best practices in optimal HC management [46]. In its charter on collaboration between health professionals, the Swiss Academy of Biomedical Sciences has highlighted the importance of clarifying competencies and responsibilities to ensure optimal patient-centered care, especially among health professionals working in the same organization [53].

Since new types of HCGs are now being incorporated in a field which was previously predominated by nurses and occupational therapists, it has become relevant to define everybody’s role as to optimize existing and future collaborative practices and discover potential missing elements. Define specific needs and explore possible strategies for improving patient-centered HC management and particularly for optimizing collaborative practice are essential conditions for improving HC, which has been little studied so far [51].

Thus, our study aims to: (1) explore existing collaborative HC practice in French-speaking Switzerland at the micro level (level that directly concerns patients and interactions with them [54]), including HCGs not employed by agencies; (2) explore and identify the roles of various HCGs (whether employed by agencies or not) around specific HC recipients in French-speaking Switzerland; (3) identify needs and expectations of all the HCGs and stakeholders in HC (including recipients) to create a clear vision of future optimal HC management; and (4) identify strategies to improve HC management and, particularly, to optimize collaborative practice.

## Method

### Theoretical framework

The study will be guided by the concepts and approaches of “patient-centered care” and “collaborative practice”, with investigations supported by “social network analysis”.

**Fig. 1:**
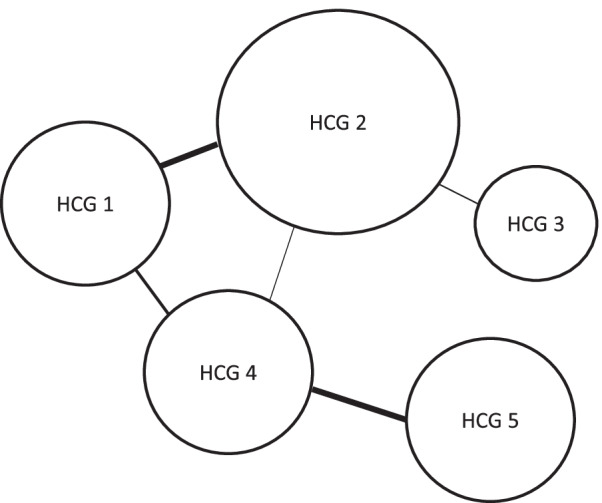
Example of a network (line thicknesses are proportional to the frequency of collaborations and node sizes are proportional to the HCG’s number of collaborations)

- The Institute of Medicine defines the patient-centered care approach as “providing care that is respectful of and responsive to individual patient preferences, needs, and values and ensuring that patient values guide all clinical decisions” (p. 40) [36].
- Collaborative practice in HC is “when multiple health workers from different professional backgrounds provide comprehensive services by working with patients, their families, carers and communities to deliver the highest quality of care across settings” [48].
- Social network analysis is an approach that creates representations of the links between various actors, and it considers that how those links are configured has consequences for the people involved [55]. One the approach’s characteristics is that the network analyzed is represented graphically [55]. Different variables are represented by nodes and their relationships by lines connecting those nodes [56]. In our study, we will represent HCGs with nodes and the collaborations between them with lines (see Fig. 1).

### Design

We will conduct a multiphase mixed-methods study with a research design focused on research questions requiring both quantitative and qualitative methods and data collection. The added value of combining both datasets will strengthen our interpretation of them [57–59]. This type of design is particularly appropriate to our research objectives. Indeed, our first objective focuses on the micro level of HC—(1) exploring existing collaborative HC practice in Switzerland by including the HCGs not employed by agencies—will require the collection of quantitative data to provide an overview of existing collaborative practice in HC in French-speaking Switzerland. Our other objectives—(2) exploring and identifying the roles of various HCGs, (3) identifying needs and expectations of all stakeholders, and (4) identifying strategies to improve HC management and, particularly, optimizing collaborative practice—will require a qualitative approach to enable us to explore these questions in depth, but fully interpreting and answering the research questions will mean combining qualitative and quantitative data.

The study will be conducted in three distinct consecutive phases [58]. This multi-phase design allows for a better and growing understanding of a phenomenon through the different research phases combining the sequential collection of quantitative and qualitative data [60]. The data integration process will follow a formally designed analytical plan [58] in which the analyzed quantitative data will form the basis of the information used in our qualitative research methods.

**Fig. 2:**
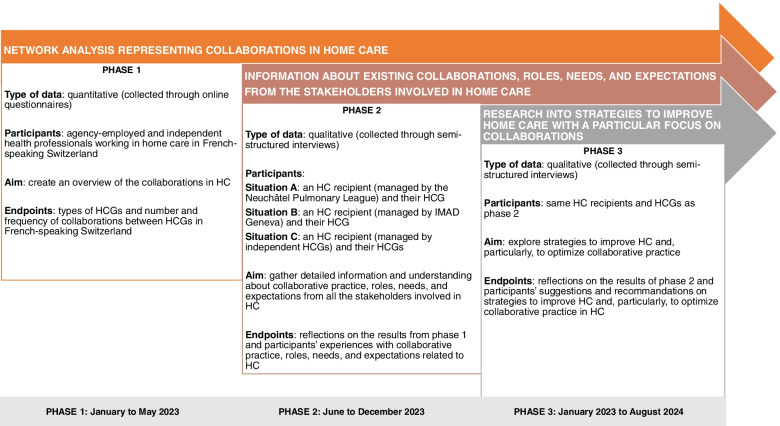
Study overview

The study’s three phases are represented in Fig. 2. In phase 1 (quantitative phase), a social network analysis [55] will give us an overview of collaborative practice in HC in French-speaking Switzerland. After completion, phase 2 (qualitative phase) will focus on investigating three different situations (A, B, and C) involving various locations (rural and urban) and HC functioning (HCGs employed by agencies and working independently). In each situation, we will conduct a semi-structured interview with a participating HC recipient and a semi-structured interview with each of their HCGs. This phase aims to discuss the results of *phase 1*’s network analysis and provide more detailed information about existing collaborative practice. The roles, needs, and expectations of stakeholders involved in HC will also be discussed. *Phase 3* (qualitative phase) will also consist of semi-structured interviews with the same participants as in *phase 2*. The aims will be to discuss *phase 2*’s findings and to explore strategies to improve HC and, particularly, to optimize collaborative practice. We opted for semi-structured interviews rather than focus groups in order to avoid any self-censorship or conforming to majority opinions [61].

### Study settings

This study will be conducted between January 2023 and December 2024. As Switzerland is a confederation of 26 cantons that organize HC differently, striving for a comprehensive national overview and highlighting each canton’s specificities would be infeasible in this relatively small study. We thus decided to focus on French-speaking Switzerland (composed of 7 cantons) in *phase 1* and then narrow the scope for *phases 2* and *3* to ensure local support and partnerships. These two phases will investigate three different situations regarding the context in complementary environments (A, B, and C), which will provide a relatively broad overview of the HC situation in French-speaking Switzerland. Situation A will examine a HC case in a rural canton run by a HC agency. Situation B will examine a HC case in an urban canton run by a HC agency. Situation C will examine a HC case managed by independent HCGs (not managed by agencies) in a canton of French-speaking Switzerland.

### Participants and recruitment

In *phase 1*, we will establish a list of the HC agencies and independent HCGs working in French-speaking Switzerland, mainly through internet research. Contacts will be made with HC agencies in our selected focus cantons. Additionally, independent HCGs will be identified through the websites belonging to professional associations (e.g., Switzerland's national associations for nurses, occupational therapists, physiotherapists, or dieticians) and associations of independent HCGs (e.g., associations of independent nurses, psychologists, physiotherapists, or GPs). We will also use our professional network to recruit the required participants. In February 2023, we will invite all the identified agencies to collaborate with the study and disseminate an internet link to our online questionnaire to their employees and any independent HCGs with whom they cooperate. We also will send an email invitation with the link to an online questionnaire developed for this purpose to all the independent HCGs identified. The aim is to include as many HCGs as possible in French-speaking Switzerland. In order to have relevant results, we would like to include a variety of different HC professionals dealing with a sample of at least 40 participating HCGs, in line with what is reported in the literature [62].

In *phase 2*, volunteers will be purposefully sampled [61] to ensure a broad diversity of participants (agency and independent HCGs; HCGs and HC recipients from one rural and one urban canton). This type of sampling is frequently used in the methodological approaches for qualitative [61] and mixed-methods [60] research to obtain of the needed information from heterogeneous populations [61, 63]. It allows for the selection of participants who have very particular experiences related to the topic of interest [60]. Recruitment and data collection will proceed concurrently until data saturation is reached, i.e., no new information seems to be retrieved. This is a procedure frequently used in qualitative methods [64, 65]. At this stage, therefore, we cannot specify how many participants will be included in phases 2 and 3 since this will depend on the data collected [64].

For situation A, we will collaborate with the Neuchâtel Pulmonary League and invite agency employees to describe an emblematic situation (i.e., a real-life typical situation that required the intervention of multiple different HCGs). We will then ask for permission to contact the HC recipient and all the HCGs who were involved in that specific HC situation, explain the study’s aim to them, and invite them to participate in a semi-structured interview. We will proceed in the same way for situation B, but in collaboration with the IMAD. For the situations A and B, we will invite HC recipients cared for by a HC agency, but who, if possible, also receive some HC from independent HCGs. For situation C, we will collaborate with independent HCGs to invite an HC recipient and all the independent HCGs involved in this specific case. To do this, we will contact HCGs from whom we obtained contact information during *phase 1*, explain *phase 2* and *3*’s aims, and invite them to contact us if they were involved in caring for a specific recipient who could be invited to participate in the study for situation C. In each situation, if the participating HC recipient also receives care from informal HCGs, they too will be invited for participation in the study. In cases where several informal HCGs provide care to the participating HC recipient, all of them will be invited for participation and interviewed when agreed.

In *phase 3*, we will interview the same participants as in *phase 2*. The number of participants in *phases 2* and *3* will depend on the number of HCGs involved in the different cases. To facilitate recruitment for HCGs regarding their time spent assisting the study, participants in *phases 2* and *3* will be compensated with CHF 50 per hour of interview.

### Data tools

To generate our network analysis, an online questionnaire will be created using REDCap software [66] and containing the following two multiple-choice questions developed by the research team to address *phase 1*’s aims: (1) Which other HCGs do you collaborate with when caring for recipients at home? (2) How often do you collaborate with each of those HCGs? In addition, at the start of the questionnaire, participants will be asked to provide demographics like gender, age, profession, and how long they have been working in HC. Before starting data collection, the online questionnaire will be tested among a small number of HCGs not involved in the research team and it will be adapted if necessary.

The online questionnaire’s results will allow us to represent existing collaborations and their frequency via a node-and-spoke diagram as shown in Fig. 1.

In *phase 2*’s semi-structured interviews, participants will be invited to state their age, occupation and how long they have been working in HC. *Phase 1*’s results will be discussed with the participants and a diagram representing all collaborations in French-speaking Switzerland will be shown. Different questions regarding collaborations, roles, needs and expectations will then be asked to the participants (see interview guide in Additional file 1: Appendix I).

The same topics will be discussed in each interview with HC recipients, but some questions will be adapted to their situation and perspective. This will also be the approach used in interviews with the informal HCGs included in the study.

Before starting the interviews, the interview guide will be tested with a small number of HCGs not involved in the research team and it will be adapted if necessary.

*Phase 3*’s semi-structured interviews will involve the same participants as *phase 2*. *Phase 2*’s outcomes—the findings from situations A, B, and C—will be discussed with them, and then we will ask about their opinions concerning each situation and whether the findings represent their daily activities and why. *Phase 3*’s interview guide will be developed using *phase 2*’s findings after those qualitative data have been analyzed.

Finally, during *phase 3*, participants will be questioned about their ideas and proposals for optimizing HC, with a particular focus on collaborative practice. As the content of these discussions will depend on *phase 2*’s outcomes, they will also be developed once the qualitative data from *phase 2* have been analyzed.

### Data collection

In *phase 1*, we will send an email with a link to the online questionnaire to all persons on the list mentioned above. *Phase 2*’s data collection will only start once all the *phase 1* data have been analyzed and interpreted. In *phase 2*, we will conduct semi-structured interviews concerning situations A, B, and C with all the HCGs and HC recipients involved in them. All interviews will be conducted and audio-recorded by a member of the research team. *Phase 3* data collection will only start once all the data from *phase 2* have been analyzed and interpreted. *Phase 3* will involve semi-structured interviews with the same participants as for *phase 2*, conducted and recorded by the same team. For each phase, data will only be collected after participants have been given the appropriate study information and have provided their written informed consent in return.

### Data processing and analysis

Part of the evaluation of *phase 1* (quantitative data collected through online questionnaires) will involve a network analysis. The online questionnaire’s results will be inserted into an Excel file, where a “1” will indicate a connection between two HCGs and a “0” will indicate no connection. These data will then be imported into R software [67], which will allow us to create the graphical representation of the network as well as the different measures related to it [68]. A strategy and an analysis plan will be developed and detailed with a biostatistician.

*Phase 2* and *3*’s audio-recordings (qualitative data) will be transcribed verbatim. We will then perform an open coding of the transcripts to express their meaning [61]. A thematic analysis will be made to identify, analyze, and report on the themes emerging from the data [69]. This will follow the steps described by Braun and Clarke [69]: familiarization with data, generation of codes, identification of themes, verification of themes, definition and naming from themes, and writing a report.

### Reliability/Credibility

To ensure the credibility of the qualitative component’s findings, interview responses will be coded. There will be a verification that no information is getting lost. In addition, some parts of the interviews will be coded by another researcher so that there can be a discussion between researchers about the selection of appropriate codes.

### Validity/Trustworthiness

To ensure the validity of the qualitative component’s findings, the data collection process, the context, and how the interviews were conducted will be described in detail. To ensure the trustworthiness of the qualitative data, interviews will be audio-recorded, transcribed in a standardized manner, and then compared with the audio-recording afterwards.

## Discussion

Over the past few decades, demographic evolution has provoked a shift from hospitalized care towards HC. This evolution has been accompanied by the fact that new types of HCGs are now intervening in a field which was in the past predominated by nurses and occupational therapists. In this context, identify the roles of each is very pertinent, as is the way they perceive their roles and how they collaborate. We are convinced that an in-depth examination of each professional’s skills and roles is fundamental to reinforcing existing collaborative practice and to establishing new collaborations that will lead to improvements in the quality of HC management and provision.

Thanks to an analysis of existing collaborations, the roles but also the expectations of HGGs and HC recipients can be illustrated to obtain a good overview of the functioning of collaborative practice in HC in French-speaking Switzerland. This will enable recommendations so that possible means can be implemented to meet the expectations of those concerned. Moreover, thanks to the last phase of the study (qualitative phase exploring strategies), HCGs and HC recipients themselves will be able to directly reflect on potential solutions to improve collaborative practice and thus the quality of care.

Although this study is limited to French-speaking Switzerland, the results will provide an opportunity for reflection that also will benefit other regions of Switzerland. Furthermore, to obtain a complete overview of the functioning of collaborative practice in HC in Switzerland, it would be relevant that the same study be conducted in the other regions of Switzerland in the near future.

In addition, the present study will be a starting point for new projects aiming to establish and test actions to enhance communication and collaborative practice in HC, and ultimately to improve the quality of care. Moreover, our study’s findings will allow to highlight which elements related to the collaborations between the different HC stakeholders need to be optimized. They will, therefore, serve as a basis for developing further studies meeting these specific needs. Furthermore, our results will provide the basis for upcoming interprofessional projects planned in the field of HC and health promotion.

Finally, this study’s findings will be an excellent complement to the study by Möckli et al. [70], which will be carried out in Switzerland at the national level. These authors will focus on the interactions between the macro-, meso-, and micro-factors in care coordination by collecting data from HC agencies [70]. They also plan to explore care coordination’s associations with the quality of care [70]. Good coordination seems to be essential for quality HC [40], so a more detailed analysis of current formal and informal collaborative practice at the micro level is essential. That level directly concerns patients and interactions with them [54], integrating not only the HCGs hired by home care agencies but also independent HCGs. However, independent or free-lance HCGs not employed by HC agencies will not be included in Möckli et al.’s study [70]. Our study, which focuses on both HCGs employed by agencies and HCGs working independently will therefore be of necessary complementary value in exploring and evaluating the strengths and limitations of HC in Switzerland, next to their research. Results of these two complementary studies will provide recommendations for the development of a Swiss collaborative interprofessional model for HC.

## Conclusion

Home care is an evolving field in which many different professionals are involved to provide the best possible care to patients. Good communication and collaboration between all the stakeholders involved is an essential condition to provide quality care. In Switzerland, however, there is a lack of data on how these collaborations are currently functioning. The results of our study will allow to collect relevant information on current collaborative practice in home care in French-speaking Switzerland, but also to explore the roles and expectations of each of the concerned stakeholders. It also will enable to propose recommendations and strategies to optimize collaborative practice in home care. Finally, it will serve as a basis for future studies that can be conducted to address the identified needs.

## Supplementary Information

**Additional file 1: Appendix I . **Interview guide.

## Data Availability

Not applicable.

## Abbreviations

HC: Home care
OA: Older adults
HCG: Home care giver
