# Supplementary material for: A social network analysis to explore collaborative practice in home care: research protocol
Source: BMC Health Serv Res. 2022 Sep 19;22:1174. doi: 10.1186/s12913-022-08548-4 (PMC9484240; doi:10.1186/s12913-022-08548-4)
Supplement: Supplementary file 1 — Additional file 1: Appendix I . Interview guide. [file 12913_2022_8548_MOESM1_ESM.docx]

**APPENDIX I**

**Interview guide**

- What do you think about the overall results of the network analysis representing all collaborations in HC in French-speaking Switzerland?
- Does this current social network representing all collaborations in HC in French-speaking Switzerland reflect your daily experience? If not, why?
- How would you define optimal HC?
- How important is collaborative practice for optimal HC?
- What types of collaboration do you have?
  - Exchange of information? If yes, how? Does it work well? If not, why not?
  - Shared decision-making? If yes, with whom? Does it work well? If not, why not?
  - Team sessions? If yes, with whom? At what frequency? Does it work well? If not, why not?
  - Treatments together with other HCGs? If yes, with whom? At what frequency? Does it work well? If not, why not?
- Should collaborations with some HCGs be improved? If yes, with which HCGs should collaborations improved? Why? How?
- How do you define your role in HC?
- How do you define the roles of other HCGs (nurses, occupational therapists, dieticians, psychologists, doctors, social workers, …) in HC?
- What would you need to provide more optimal HC (more time, more money, more resources, a coordinator, ...)?
- What are your expectations of your needs being met?
